# Sleep Power Spectral Density and Spindles in PTSD and Their Relationship to Symptom Severity

**DOI:** 10.3389/fpsyt.2021.766647

**Published:** 2021-11-19

**Authors:** Dan Denis, Ryan Bottary, Tony J. Cunningham, Shengzi Zeng, Carolina Daffre, Kaitlyn L. Oliver, Kylie Moore, Samuel Gazecki, Augustus Kram Mendelsohn, Uriel Martinez, Karen Gannon, Natasha B. Lasko, Edward F. Pace-Schott

**Affiliations:** ^1^Department of Psychology, University of Notre Dame, Notre Dame, IN, United States; ^2^Department of Psychology and Neuroscience, Boston College, Chestnut Hill, MA, United States; ^3^Division of Sleep Medicine, Harvard Medical School, Boston, MA, United States; ^4^Department of Psychiatry, Massachusetts General Hospital, Charlestown, MA, United States; ^5^Department of Psychiatry, Beth Israel Deaconess Medical School, Boston, MA, United States; ^6^Department of Psychology, The University of Hong Kong, Pokfulam, Hong Kong SAR, China; ^7^Department of Psychiatry, Harvard Medical School, Charlestown, MA, United States; ^8^Department of Neurology, Massachusetts General Hospital, Charlestown, MA, United States; ^9^Athinoula A. Martinos Center for Biomedical Imaging, Charlestown, MA, United States

**Keywords:** post-traumatic stress disorder, sleep, spectral power, sleep spindles, beta power

## Abstract

Sleep disturbances are common in post-traumatic stress disorder (PTSD), although which sleep microarchitectural characteristics reliably classify those with and without PTSD remains equivocal. Here, we investigated sleep microarchitectural differences (i.e., spectral power, spindle activity) in trauma-exposed individuals that met (*n* = 45) or did not meet (*n* = 52) criteria for PTSD and how these differences relate to post-traumatic and related psychopathological symptoms. Using ecologically-relevant home sleep polysomnography recordings, we show that individuals with PTSD exhibit decreased beta spectral power during NREM sleep and increased fast sleep spindle peak frequencies. Contrary to prior reports, spectral power in the beta frequency range (20.31–29.88 Hz) was associated with reduced PTSD symptoms, reduced depression, anxiety and stress and greater subjective ability to regulate emotions. Increased fast frequency spindle activity was not associated with individual differences in psychopathology. Our findings may suggest an adaptive role for beta power during sleep in individuals exposed to a trauma, potentially conferring resilience. Further, we add to a growing body of evidence that spindle activity may be an important biomarker for studying PTSD pathophysiology.

## Introduction

Post-traumatic stress disorder (PTSD) is an emotional disorder characterized by the persistence of heightened reactivity 1 month or more after exposure to a traumatic event. Symptoms include intrusions, avoidance behaviors, and hyperarousal (1). Sleep disturbances are extremely common in PTSD, present in ~70% of patients (2). Two meta-analyses have identified polysomnographic (PSG) findings common across multiple studies of PTSD including decreased total sleep time (TST) and sleep efficiency (SE) and increased wake time after sleep onset (WASO) (3), increased light (N1) sleep and reduced slow-wave (N3) sleep (3, 4), and increased rapid eye movement density (4). Certain moderators (e.g., age, sex) also reveal subgroup specific features relative to controls and illustrate the heterogeneity but also the ubiquity of sleep disturbance in individuals with PTSD (4, 5). However, reliable microarchitectural markers of PTSD during sleep remain elusive (6). This is of critical importance as sleep macroarchitecture (i.e., time spent in different sleep stages) only provides a broad characterization of the sleeping brain that varies widely from night-to-night, potentially hindering their utility as reliable biomarkers of sleep in PTSD (7).

The quantification of sleep electroencephalography (EEG) signals through spectral power analysis allows for a more fine grained analysis of frequency-specific neural activity that is reflective of underlying brain states. Emerging evidence suggests that PTSD patients exhibit a reduction in low frequency (slow oscillation-delta band range; <4 Hz) power during non-rapid eye movement (NREM) sleep, and increased high frequency power (beta and gamma frequency range; >20 Hz) during both NREM and REM sleep (6, 8–12), though see (13, 14). In addition, trauma exposed individuals who do not go on to develop PTSD have been found to show higher REM theta (4–7 Hz) power compared to patients who did subsequently develop PTSD (15). These findings are hypothesized to indicate lower restorative functioning and increased hyperarousal during sleep in PTSD.

Another sleep oscillation that has been suggested as a potential pathophysiological biomarker are sleep spindles. These waxing and waning (~9–16 Hz) oscillations are characteristic signatures of NREM sleep (16). Generated by the thalamic reticular nucleus (TRN), spindles travel to cortical regions via thalamocortical pathways (17, 18). A number of putative functions of sleep spindles have been suggested, including sleep maintenance, induction of synaptic plasticity, and memory consolidation [see (17) for detailed review]. Given their relatively well-understood generation by thalamo-cortical circuitry, scalp-EEG detected sleep spindles may provide an indirect, non-invasive readout of thalamocortical functioning during sleep (19). Unlike sleep macroarchitecture, sleep spindle properties are highly stable night-to-night, showing high test-retest reliability (20). Surprisingly, only two studies to date have examined sleep spindle activity in PTSD (6, 21), with one study suggesting a higher sleep spindle peak frequency in PTSD patients (21).

The aim of the present study was to compare NREM and REM sleep microarchitecture in PTSD patients to trauma-exposed individuals who did not go on to develop clinically diagnosed PTSD. We focused on differences in the NREM and REM power spectrum, and properties of sleep spindles. These were examined both in terms of group differences in sleep microarchitecture and correlations between sleep microarchitecture measures and psychiatric symptomatology. Our pre-registered hypotheses (https://osf.io/z6gfw) were as follows:

H1: PTSD patients would exhibit increased high frequency (>20 Hz) and decreased low frequency (<4 Hz) NREM spectral power compared to trauma-exposed controls (TEC).
H2: PTSD patients would exhibit increased high frequency (>20 Hz) and decreased theta (4–7 Hz) REM spectral power compared to TEC.
H3: PTSD patients would show higher frequency sleep spindles during NREM sleep compared to TEC.

Further exploratory analyses are described in sections below.

## Methods

We analyzed data from an existing dataset examining sleep and neuroimaging correlates of fear extinction learning in trauma exposed individuals. All reported analyses are novel, and the analysis plan was pre-registered on Open Science Framework (https://osf.io/z6gfw).

### Participants

A total of 133 right-handed participants from the greater Boston metropolitan area were recruited via online and posted advertisements and passed initial telephone screening and had satisfied inclusion/exclusion criteria following clinical interviews (22, 23). All participants had experienced a DSM-V criterion-A traumatic event (“index trauma”) within 1 month to 2 years of study participation. Current and lifetime histories of psychiatric disorders were assessed using the Structured Clinical Interview for DSM-IV-TR for Non-Patients [SCID 1/NP; (24)]. PTSD was diagnosed by a highly experienced interviewer (N.B.L.) using the Clinician-Administered PTSD Scale for DSM-5 [CAPS-5; (25)]. The PTSD Checklist for DSM-5 [PCL-5; (26)] was used to further assess symptom severity. In alignment with the National Institutes of Mental Health Research Domain Criteria (RDoC) Framework (27, 28), our study aimed to recruit participants across a range of post-traumatic symptomatology, including those with concurrent anxiety and depressive disorders. However, participants could not have a lifetime history of psychosis, bipolar disorder, autism spectrum or other neurodevelopmental disorder, suicide attempt or current or chronic suicidal ideation; neurologic conditions such as seizure, neurodegenerative disease, stroke, or other brain lesions; history of head trauma with concurrent loss of consciousness; severe medical conditions including cardiovascular or other systemic disease, chronic pain, or endocrine disorders. Participants could not have a current diagnosis of disorders known to influence sleep such as fibromyalgia, severe GERD, or chronic fatigue syndrome. However, participants were not excluded if they indicated a lifetime history of DSM-IV-TR Primary Insomnia, Insomnia Related to Another Mental Disorder or Nightmare Disorder. Participants could not meet criteria for current drug or alcohol abuse or dependence. To confirm presence of current drug use, participants completed a urine test, which identified commonly abused drugs (cocaine, THC, opiates, amphetamines, MDMA, PCP, benzodiazepines, barbiturates, methadone, tricyclic antidepressants, oxycodone, and buprenorphine). Participants treated with antidepressants were required to be on a stable dose and were accepted into the study on a case-by-case basis in consultation with the study physicians.

Of the 133 study participants, 68 participants met criteria for PTSD based on their CAPS-5 score. Sleep disorders were screened using the Pittsburgh Structured Clinical Interview for Sleep Disorders (SCID-SLD), a widely used (29, 30), but unpublished, in-house instrument. Lastly, participants completed a urine toxicology screening for 11 abused substances. The present analysis included 114 participants who successfully completed a baseline night of polysomnographically-recorded sleep (see Procedures below). Of these participants, 17 were excluded due to having unusable EEG data (e.g., excessive noise, recording error, corrupted file). As such, a total of 97 participants (52 TEC, 45 PTSD) were included in this analysis. Participant characteristics are displayed in Table 1.

**Table 1:** Sample characteristics.

	**TEC (*n* = 52)**	**PTSD (*n* = 45)**	**Sig**	**Effect size**
**DEMOGRAPHICS**				
Age (years)	23.8 (4.79)	24.2 (4.80)	0.66	0.09
**Sex**				
Female	28 (54%)	36 (80%)	**0.007**	
Male	24 (46%)	9 (20%)		0.25
**Race**				
American Indian or Alaskan Native	1 (2%)	2 (4%)		
Asian	6 (12%)	5 (11%)		
Black or African American	9 (17%)	7 (16%)		
More than one race	4 (8%)	5 (11%)		
White	30 (58%)	26 (58%)		
Prefer not to say/unreported	2 (4%)	0 (0%)		
**Ethnicity**				
Hispanic or Latino	3 (6%)	8 (18%)		
Not Hispanic or Latino	46 (88%)	37 (82%)		
Prefer not to say/unreported	3 (6%)	0 (0%)		
**Marital status**				
Married	4 (8%)	2 (4%)		
Separated	0 (0%)	1 (2%)		
Single	44 (84%)	40 (89%)		
Prefer not to say/unreported	4 (8%)	2 (4%)		
**Highest level of education**				
High school degree or equivalent	3 (6%)	0 (0%)		
Associate's degree	3 (6%)	1 (2%)		
Some college	22 (43%)	16 (36%)		
Bachelor's degree	15 (29%)	16 (36%)		
Graduate degree	4 (8%)	10 (22%)		
Prefer not to say/unreported	5 (10%)	2 (4%)		
**Income**				
< $20,000	10 (19%)	9 (20%)		
$20,000–$34,999	10 (19%)	10 (22%)		
$35,000–$49,999	7 (14%)	1 (2%)		
$50,000–$74,999	6 (12%)	5 (11%)		
$75,000–$99,999	3 (6%)	5 (11%)		
$100,000–$149,999	2 (4%)	3 (7%)		
$150,000–$199,999	1 (2%)	1 (2%)		
$200,000 +	6 (12%)	0 (0%)		
Prefer not to say/unreported	6 (12%)	6 (13%)		
**Employment status**				
Employed (1–39 h per week)	21 (40%)	20 (44%)		
Employed (40+ h per week)	14 (27%)	15 (33%)		
Not employed, looking for work	1 (2%)	2 (4%)		
Not employed, not looking for work	1 (2%)	1 (2%)		
Unemployed, looking for work	6 (12%)	3 (7%)		
Unemployed, not looking for work	4 (8%)	1 (2%)		
Prefer not to say/unreported	5 (10%)	3 (7%)		
**PTSD SEVERITY**				
Months from trauma to study	12.5 (6.99)	12.4 (6.34)	0.93	0.02
CAPS total	11.0 (6.31)	31.8 (7.87)	**<0.001**	2.92
CAPS hyperarousal	2.94 (2.53)	9.0 (3.36)	**<0.001**	2.04
PCL-5 total	18.1 (9.89)	40.5 (12.6)	**<0.001**	1.98
PCL-5 hyperarousal	3.35 (2.63)	8.51 (3.75)	**<0.001**	1.60
**PSYCHOPATHOLOGY MEASURES**				
DASS	19.4 (17.5)	47.0 (23.6)	**<0.001**	1.32
DERS	109 (19.6)	85.4 (25.3)	**<0.001**	1.03
HVQ	22.7 (10.2)	32.3 (12.1)	**<0.001**	0.86
**RETROSPECTIVE SLEEP MEASURES**				
PSQI	5.68 (2.66)	8.34 (3.18)	**<0.001**	0.91
PSQI PTSD	4.22 (4.28)	6.95 (3.78)	**0.002**	0.68
ESS	6.66 (3.43)	9.14 (4.94)	**0.007**	0.58
MEQ	48.4 (8.79)	44.1 (9.24)	**0.02**	0.48
**SLEEP DIARY MEASURES**				
Total sleep time (min)	451 (56.7)	441 (59.7)	0.40	0.18
Sleep onset latency (min)	20.3 (13.9)	28.6 (18.9)	**0.021**	0.50
Sleep efficiency (%)	92.8 (6.50)	90.1 (6.12)	**0.037**	0.44
Number of nightmares	0.64 (1.03)	1.34 (1.90)	**0.033**	0.46
**ACTIGRAPHY MEASURES**				
Total sleep time (min)	423 (59.9)	429 (66.0)	0.64	0.10
Sleep onset latency (min)	32.3 (35.5)	26.7 (19.4)	0.35	0.20
Sleep efficiency (%)	86.5 (7.46)	87.3 (8.26)	0.63	0.10
**BASELINE NIGHT POLYSOMNOGRAPHY MEASURES**				
Total sleep time (min)	384 (105)	336 (136)	0.062	0.39
Sleep onset latency (min)	25.5 (32.8)	25.6 (39.7)	0.99	<0.01
Sleep efficiency (%)	86.7 (9.26)	83.4 (14.2)	0.23	0.27
N1 %	6.85 (3.88)	5.11 (3.35)	**0.020**	0.48
N2 %	57.0 (7.42)	52.4 (13.0)	**0.042^a^**	0.43
N3 %	18.6 (9.56)	25.5 (16.7)	**0.017^a^**	0.51
REM %	17.9 (6.33)	17.4 (8.32)	0.74	0.07

*All values reflect the mean (standard deviation) with the exception of sex through employment status, where frequency (percentage) are displayed. Significant differences between groups assessed using independent samples t-test with Cohen's d used as the measure of effect size, with the exception of sex, where differences were assessed using a chi-square test and Cramer's V measure of effect size. CAPS, Clinician Administered PTSD Scale; Higher score, greater symptomatology; PCL, PTSD checklist (self-report); Higher score, greater symptomatology; DASS, Depression Anxiety Stress Scales; A higher score, greater negative emotional states; DERS, Difficulties in Emotion Regulation Scale; A higher score, better able to regulate negative emotions; HVQ, hypervigilance questionnaire; A higher score, increased hypervigilance; PSQI, Pittsburgh Sleep Quality Index; A higher score indicates poorer overall sleep quality. PSQI PTSD, Pittsburgh Sleep Quality Index for Post-traumatic Stress Disorder; A higher score, higher frequency of sleep disturbances common to PTSD; ESS, Epworth Sleepiness Scale; A higher score, increased daytime sleepiness; MEQ, Morning Evening Questionnaire; A higher score indicates greater preference for mornings. Bold values indicate cases where p < 0.05 (uncorrected)*.

a *Group difference was not significant after controlling for total sleep time*.

### Procedure

Participants completed a ~2-week sleep assessment period during which they continuously wore an Actiwatch-2 (Philips Respironics, Bend, OR) and filled out a sleep and nightmare diary. Over this period, participants also completed an online battery of questionnaires assessing trauma history, habitual sleep quality, circadian preference, anxiety, mood, and personality variables using the Research Electronic Data Capture (REDCap™) system (© 2013, Vanderbilt U). Approximately midway through the assessment period, participants completed a combined sleep-disorders-diagnostic and acclimation night of ambulatory polysomnography (PSG). This was followed by a second night of PSG recording (“baseline night”), a night prior to participants completing a fear conditioning and extinction protocol during functional magnetic resonance imaging followed by a third night of PSG-recorded sleep [see (23)]. Alcohol and recreational drugs were prohibited throughout the protocol. In the present analysis we focus solely on the baseline night of PSG. We chose to use this night for our analysis to avoid potential first-night effects as participants became acclimated to the PSG device during the acclimation/diagnostic night and the influence of learning on sleep physiology, as has been previously reported (31–33).

### Ambulatory Polysomnography

Participants underwent three nights of ambulatory polysomnography (PSG) wearing the Somte-PSG recorder (Compumedics USA, Inc., Charlotte, NC). Electrodes were attached in the laboratory and participants were sent home to sleep. The montage included 6 EEG channels (F3, F4, C3, C4, O1, O2) with reference to contralateral mastoids (M1, M2), 2 electrooculogram (EOG) channels, 2 submental electromyogram (EMG) channels, and 2 electrocardiogram (ECG) channels (right clavicle and left 5th intercostal space). The first sleep recording was considered an acclimation and sleep-disorders screening night and included additional channels for respiration transducer belts, pulse-oximeter, nasal cannula, and tibialis movement sensors. Signals were recorded at 256 Hz, using high (0.16 Hz), and low (102 Hz) pass filters. Records were subsequently exported, with a 0.3–35 Hz band pass filter (plus 60 Hz notch filter) for sleep scoring. Records from the baseline PSG night (the focus of this analysis) were scored in 30-s epochs by an experienced, Registered Polysomnographic Technologist (K.G.) following American Academy of Sleep Medicine criteria (34). The acclimation night was examined by the same scorer for clinically significant obstructive sleep apnea and periodic limb movement disorder, though no participants in the current study met clinical criteria for a diagnosis of either of these disorders. The scored records from the baseline night were used to obtain sleep macroarchitecture measures (Table 1), and were exported as European Data Format files for further analysis of sleep microarchitecture. All subsequent analyses were carried out in MATLAB using custom code (35).

### Artifact Detection

Artifactual epochs of PSG data were detected using an automated algorithm. For each EEG channel we calculated per-epoch summary metrics of three Hjorth parameters [signal activity, mobility, and complexity; (36)], and any epochs in which at least 1 channel was >3 standard deviations from the mean on any of the three parameters were marked as artifact and removed from subsequent analysis (37). Artifact detection was performed twice (in case of extreme outlying epochs), and performed separately for each sleep stage (given the inherent differences in the EEG signal between different sleep stages).

### Power Spectral Density

Estimates of power spectral density (PSD) were obtained for both NREM (N2 + N3) and REM sleep at frontal (F3, F4) and central (C3, C4) electrodes. PSD was estimated using Welch's method with 5 s Hamming windows and 50% overlap. To minimize 1/f scaling, PSD estimates were derived from the derivative of the EEG time series (20). PSD estimates at each channel and sleep stage were then normalized within-subject by dividing power at each frequency between 0 and 30 Hz by the average power in the 0–30 Hz range [see (38) for a similar approach]. PSD estimates at the two frontal sites were averaged together to obtain a measure of frontal PSD activity, and PSD estimates at the two central sites were averaged together to obtain a measure of central PSD activity.

### Sleep Spindles

Sleep spindles were automatically detected at frontal and central electrodes during NREM sleep using a previously-validated wavelet based detector (35, 39, 40). As a first step, individualized fast and slow spindle peak frequencies were identified through visual inspection of the NREM sleep power spectrum (using both frontal and central electrode sites, with PSD estimated using the same procedure described above). An individual's fast spindle peak was defined as the most prominent peak between 12.5 and 16 Hz, and a slow spindle peak was defined as the most prominent peak between 9 and 12.5 Hz. Any spindle peaks occurring at exactly 12.5 Hz were considered fast spindles. Detection of spindle peaks was performed blind to the patient group. Three participants did not show a clearly identifiable fast spindle peak, and 24 participants failed to exhibit a clearly defined slow spindle peak. Because the lack of a peak in the power spectrum does not necessarily indicate the lack of oscillatory activity (if those events are infrequent or of low amplitude), participants without a fast peak frequency had their fast spindle peak frequency set to 14 Hz, and participants without a slow peak frequency had their slow peak frequency set to 11 Hz. A sensitivity analysis, where all spindle based analyses were re-run excluding participants without a discernible peak, led to the same pattern of results (not reported).

After deriving each participant's individualized fast and slow spindle peaks, spindles were automatically detected using a wavelet-based detector. The raw, artifact-free, NREM EEG signal was subjected to a time-frequency decomposition using complex Morlet wavelets. The peak frequency of the wavelet was set to each individual's fast or slow spindle peak. In order to minimize overlap between fast and slow spindle ranges, the full-width half-max bandwidth was set as a 1.3 Hz window centered on the peak spindle frequency (35). Spindle detection was performed on the squared wavelet coefficients after being smoothed with a 100 ms moving average. A spindle was detected whenever the wavelet signal exceeded a threshold of six times the median signal amplitude for at least 400 ms (35, 41). Spindle detection was performed twice, once for fast spindles, and again for slow spindles.

For fast spindle analysis, fast spindle activity at central electrodes were averaged together for subsequent analyses. For slow spindles, activity at frontal electrodes were averaged. For both spindle types, the following parameters were extracted: Peak frequency (Hz), density (spindles/min), amplitude (μV), and duration (seconds).

### Age Correction

Age can act as a confounding variable in sleep macro- and microarchitecture studies (42). Most notably, increasing age is linked to a reduction in slow wave sleep, and thus a reduction in low frequency spectral power. During study recruitment, an upper age limit of 40 was selected to ensure participants retained a measurable amount of slow wave sleep. However, because the TEC and PTSD groups were not strictly age-matched, we tested for associations between age and our main sleep measures of interest (NREM spectral power, REM spectral power, and sleep spindle frequency). In all cases, no significant associations were observed between age and key sleep metrics (all *r*'s < 0.16, all *p*'s > 0.11).

### Clinical Measures

*Clinician-Administered PTSD Scale for DSM-5 (CAPS-5)*. The CAPS-5 (25) is the “gold standard” clinical assessment for PTSD. Administration of the CAPS-5 involves clinician ratings for each of the 20 symptoms of PTSD on a 5-point severity scale ranging from 0 (absent) to 4 (extreme). Total scores range from 0 to 80.

*The PTSD Checklist for DSM-5 (PCL-5)*. The PCL-5 (26) is a self-report scale that includes 20 questions based on the DSM-5 diagnostic criteria for PTSD. Participants rated symptom severity on 5-point scales. PCL-5 hyperarousal was the combined hyperarousal (Cluster E) PTSD symptoms on the PCL-5 excluding sleep item 20 (“Trouble falling or staying asleep”).

*Depression and Anxiety Stress Scale (DASS)*. The DASS (43) is a 42-item self-report scale which measures the three related negative emotional states of depression, anxiety and tension/stress.

*Difficulties in Emotion Regulation Scale (DERS)*. The DERS (44) is a 36-item self-report scale which measures subjective emotion regulation by asking respondents to identify the frequency in which participants engage in adaptive and maladaptive emotion regulation strategies using a 5 point scale from 5 (almost never) to 1 (almost always). In this dataset, the DERS was reverse scored meaning a higher score indicates less difficulty regulating emotions.

*Hypervigilance Questionnaire (HVQ)*. The HVQ (45) is an 11-item assessment of subjective hypervigilance in which participants rate items on a scale from 1 (not at all true) to 5 (*extremely true*) about their experience of hypervigilance symptoms.

*Pittsburgh Sleep Quality Index (PSQI)*. The PSQI (46) is a 19 item self-report questionnaire that assesses several different aspects of sleep quality over a 1-month period including subjective sleep quality, sleep latency and duration, sleep efficiency, sleep disturbances, use of sleep medication, and daytime dysfunction.

*Pittsburgh Sleep Quality Index for Post-traumatic Stress Disorder (PSQI PTSD)*. The PSQI PTSD (47) is a self-report instrument designed to assess the frequency of seven disruptive nocturnal behaviors including anxious nighttime thoughts and experiencing or acting out nightmares.

*Epworth Sleepiness Scale (ESS)*. The ESS (48) is an 8-item self-report questionnaire in which respondents are asked to rate their usual chances of dozing off or falling asleep while engaged in different activities. Ratings are made on a 4-point scale from 0 (would never doze) to 3 (high chance of dozing).

*Morning Evening Questionnaire (MEQ)*. The MEQ (49) is a 19-item self-report measure that identifies individuals' preference for a morning, evening or intermediate chronotype. Questions include preferred sleep and wake times and preferred times for daytime activities.

### Statistical Analysis

Our *a priori* planned analyses can be found in the study pre-registration (https://osf.io/z6gfw). Group differences in PSD between TEC and PTSD were assessed using cluster-based permutation tests implemented in the FieldTrip toolbox for MATLAB (50). This approach allowed us to take the full power spectrum into account whilst also accounting for multiple comparisons. The *ft_statfun_indsamplesT* function was used with the following parameters: 10,000 iterations, a *clusteralpha* of 0.1 with the default *maxsum* method to determine cluster significance, and a significance threshold of 0.05. Separate tests were run for NREM and REM PSD. To test for significant associations between spectral power and symptomatology, our pre-registered *a priori* approach was to average PSD across each frequency in significant clusters to obtain a single value that could then be correlated with PCL hyperarousal score. In the absence of any significant group-differences, our unplanned *post-hoc* approach was to perform the spectrum-wide correlations with symptomatology utilizing the same cluster-based permutation approach, though with the *ft_statfun_correlationT* function. In this report, we focus on PSD at central electrode sites. Sensitivity analyses (not reported) using the frontal electrode sites revealed a highly similar pattern of results. Group differences in fast and slow spindle frequency was assessed using two independent samples *t*-tests.

As additional planned exploratory tests, we assessed group differences and symptomatology correlations for PSD estimates obtained separately for N2 and N3 sleep, and also for early and late NREM/REM periods. Here, early NREM/REM was defined as NREM or REM sleep occurring in the first half of the night. Late NREM/REM was defined as NREM or REM sleep occurring in the second half of the night. Similarly, we also examined group differences and symptomatology correlations for sleep spindle frequency during N2 and N3 sleep separately, as well as during early and late NREM sleep. Finally, exploratory analyses of group differences in spindle density, amplitude, and duration were performed, as well as exploratory correlations between these spindle properties and symptomatology. Additional, unplanned exploratory tests are highlighted in the results section as relevant.

## Results

### Participant Characteristics

Demographic, psychometric, and sleep characteristics of the sample of participants included in this analysis are displayed in Table 1. There was no difference in age between the two groups [*t*_(87.2)_ = 0.02, *p* = 0.98, *d* < 0.01], however there was a difference in sex between groups [$X_{\left(1\right)}^{2}$ = 6.66, *p* = 0.010, *V* = 0.25]. Sleep diary reported sleep onset latency was significantly shorter in the TEC group [*t*_(75.9)_ = 2.37, *p* = 0.020, *d* = 0.50], and diary reported sleep efficiency was significantly greater [*t*_(90.9)_ = 2.12, *p* = 0.037, *d* = 0.44] compared to the PTSD group. The frequency of nightmares during the diary reporting period was significantly higher in the PTSD group [*t*_(64.1)_ = 2.18, *p* = 0.033, *d* = 0.46]. The two groups did not differ on any actigraphy measures. On the baseline PSG night (the focus of this report), N1 and N2 percentage was both significantly greater in TEC participants (*p*'s <0.043, *d'*s > 0.42). Conversely, N3 percentage was significantly greater in the PTSD group [*t*_(68.1)_ = 2.44, *p* = 0.017, *d* = 0.51]. However, when controlling for total sleep time, only the difference in N1 percentage remained significant (*p* = 0.038). Sleep onset latency and sleep efficiency were also equivalent between groups (all *p*'s > 0.30).

### Power Spectral Density

**Figure 1:**
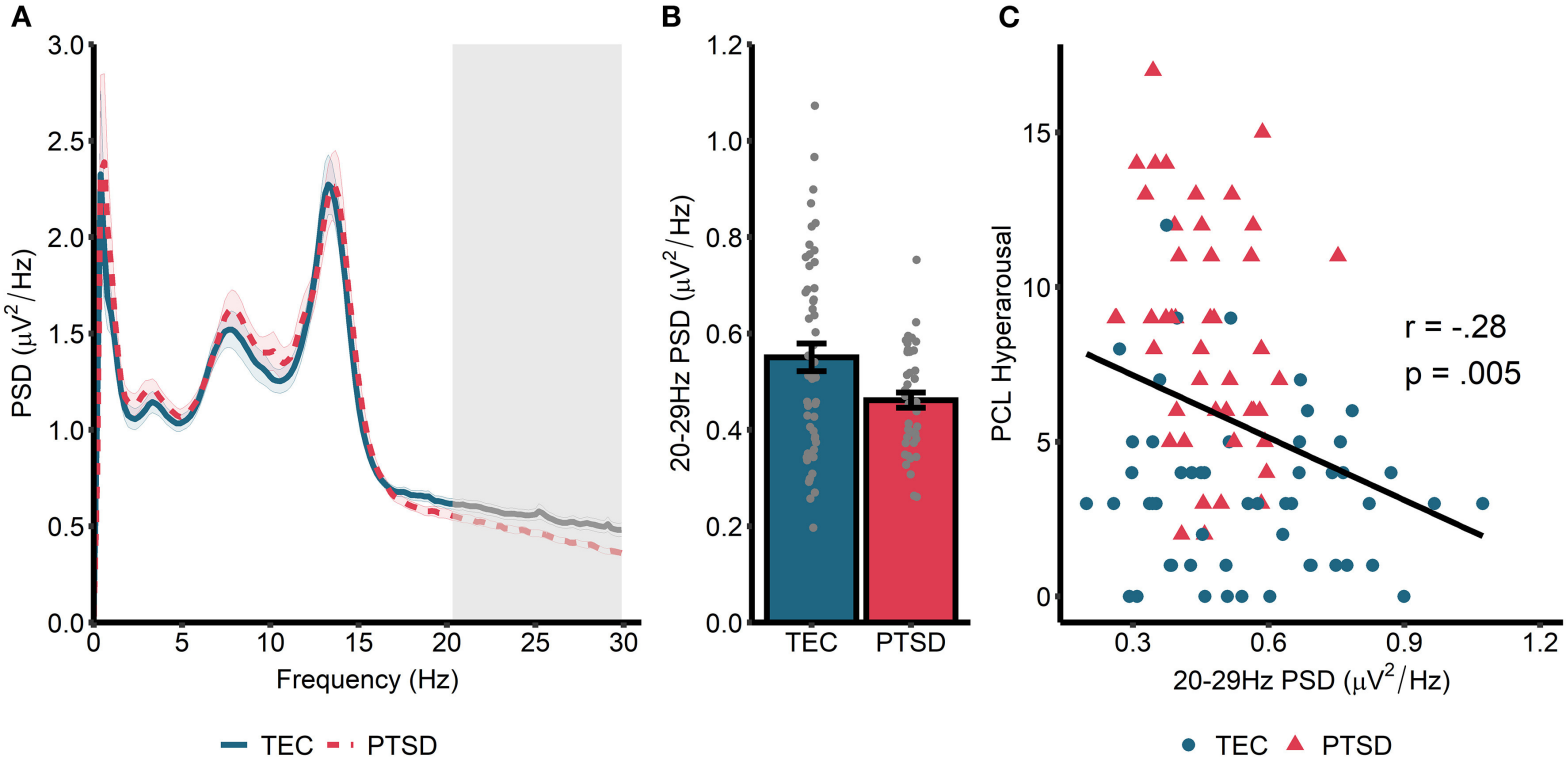
Power spectral density during NREM sleep. **(A)** 0–30 Hz power spectrum during NREM sleep. Note that PSD at each frequency is relative to the average PSD in the 0–30 Hz range. Shaded area around lines denotes the standard error. Gray box marks frequencies showing significant group differences following cluster correction. **(B)** PSD averaged across frequencies in the significant cluster. **(C)** Correlation between spectral power (averaged across frequencies in the significant cluster in **A**) and symptoms of hyperarousal.

Spectral power during non-rapid eye movement (NREM) sleep is displayed in Figure 1A. A cluster-based permutation test revealed a significant group difference in spectral power at 20.31–29.88 Hz (*t*_sum_ = 122.77, *p* = 0.015, *d* = 0.56). Spectral power in this band was higher in TEC participants (*M* = 0.55, *SD* = 0.03) compared to PTSD patients (*M* = 0.46, *SD* = 0.02; Figure 1B). As planned exploratory analyses, we tested whether these group differences would be seen in both N2 and N3 sleep, and in both early and late NREM sleep. For N2 sleep, we observed a similar significant cluster differentiating groups in frequencies spanning 24.41–29.88 Hz (*t*_sum_ = 75.07, *p* = 0.034). No significant clusters differentiating groups emerged for N3 sleep PSD. Conversely, when we compared early (which typically contains more N3 sleep) and late (which typically contains more N2 sleep) NREM, a non-significant cluster emerged differentiating groups during early NREM (23.63–29.88 Hz; *t*_sum_ = 70.80, *p* = 0.056). A significant cluster differentiating groups emerged during late NREM sleep (23.24–29.88 Hz; *t*_sum_ = 98.21, *p* = 0.018). In all cases, TEC participants showed increased spectral power relative to PTSD participants.

Next, we correlated cluster-averaged spectral power with PCL hyperarousal symptomatology (Figure 1C). Across all participants, we observed a significant *negative* correlation (*r* = −0.27, *p* = 0.008), indicating that increased 20–30 Hz power was associated with fewer hyperarousal symptoms. Correlations were not significant when run separately within the TEC (*r* = −0.13, *p* = 0.34) and PTSD (*r* = −0.23, *p* = 0.14) groups. A similar magnitude correlation was found when the total PCL was used (all participants: *r* = −0.20, *p* = 0.047; TEC: *r* = 0.06, *p* = 0.68; PTSD: *r* = −0.17, *p* = 0.27), and also when correlated with CAPS-measured hyperarousal (all participants: *r* = −0.27, *p* = 0.008; TEC: *r* = −0.13, *p* = 0.37; PTSD: *r* = −0.13, *p* = 0.42) and CAPS total score (all participants: *r* = −0.26, *p* = 0.01; TEC: *r* = −0.08, *p* = 0.56; PTSD: *r* = −0.11, *p* = 47).

**Figure 2:**
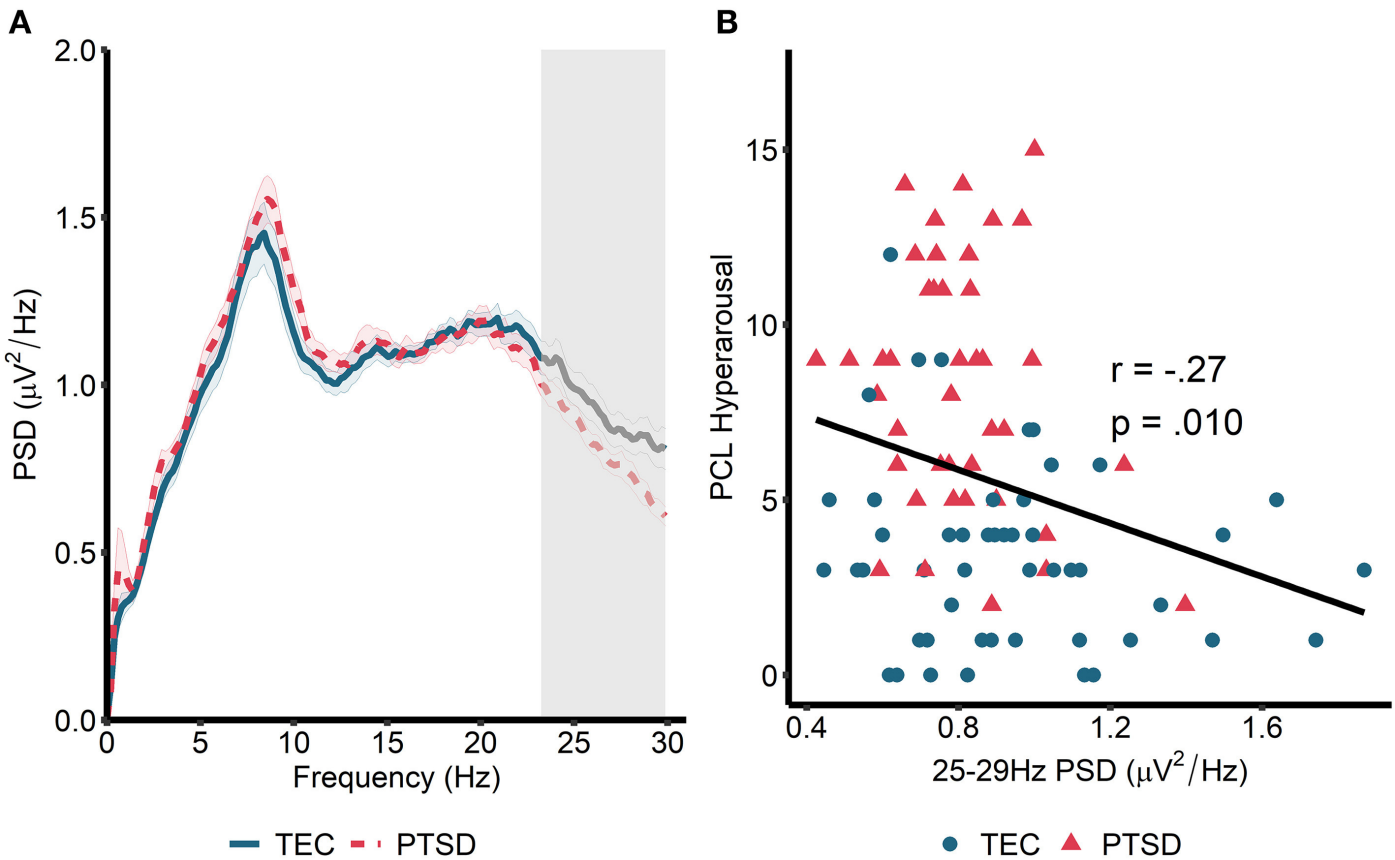
Power spectral density during REM sleep. **(A)** 0–30 Hz power spectrum during REM sleep. Note that PSD at each frequency is relative to the average PSD in the 0–30 Hz range. Gray box marks frequencies showing significant correlations with hyperarousal symptomatology across all participants following cluster correction. Shaded area around lines denotes the standard error. Note that no overall group differences were observed. **(B)** Scatterplot visualizing the relationships between cluster-averaged spectral power and hyperarousal symptomatology.

We next turned our attention to rapid eye movement (REM) sleep (Figure 2A). For REM sleep, no significant clusters emerged when we compared the REM power spectrum between the TEC and PTSD groups (all *p* > 0.23). Similarly, no group differences were found when groups were compared during early and late REM sleep separately (all *p* > 0.21). As such, we performed spectrum-wide correlations across all participants, again using a cluster-based permutation test. Here, we found a significant *negative* correlation between spectral power in a band ranging from 23.24 to 29.88 Hz and PCL measured hyperarousal (*t*_sum_ = −81.35, *p* = 0.026, cluster-averaged *r* = −0.27; Figures 2A,B). This same pattern was observed during early (25–29.88 Hz; *t*_sum_ = −64.89, *p* =0.042) but not late (*p* = 0.18) REM sleep. As with NREM sleep, correlations were not significant when run within the two groups separately (TEC: *r* = −0.17, *p* = 0.34; PTSD: *r* = −0.22, *p* = 0.18). The pattern of results remained unchanged when using the PCL total score (all participants: *r* = −0.22, *p* = 0.035; TEC: *r* = −0.08, *p* = 0.58; PTSD: *r* = −0.14, *p* = 0.41) and when using CAPS-measured hyperarousal (all participants: *r* = −0.28, *p* = 0.007; TEC: *r* = −0.18, *p* = 0.20; PTSD: *r* = −0.22, *p* = 0.18) and CAPS total score (all participants: *r* = −0.30, *p* = 0.004; TEC: *r* = −0.20, *p* = 0.16; PTSD: *r* = −0.25, *p* = 0.12). These results suggest a pattern of increased spectral power in high (>20 Hz) frequencies being associated with reduced PTSD symptomatology.

**Figure 3:**
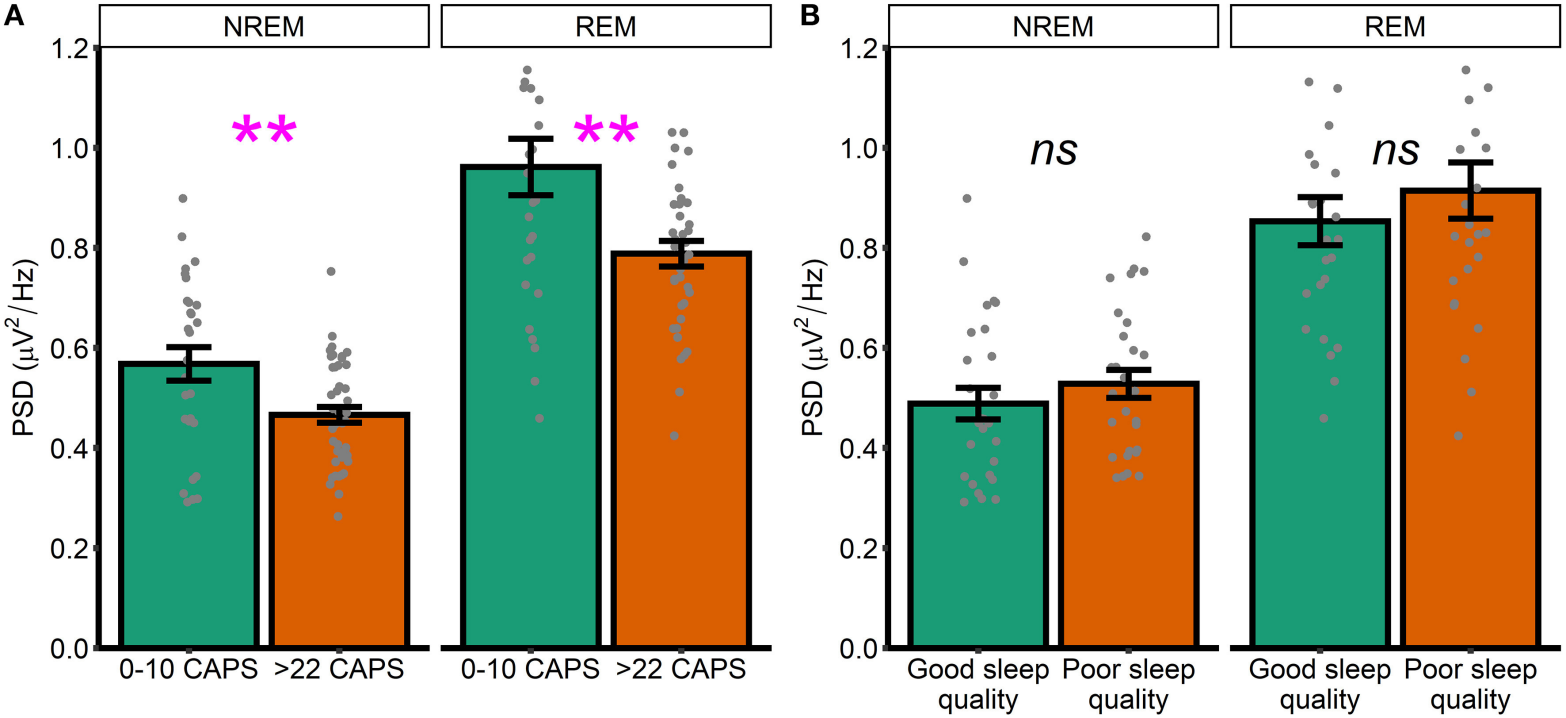
High and low symptomatology participants. **(A)** High frequency spectral power in low and high PTSD symptomatology participants, as defined by CAPS scores. **(B)** High frequency spectral power participants with good and poor sleep quality, as defined by global PSQI score. Note that PSD at each frequency is relative to the average PSD in the 0–30 Hz range Error bars = standard error. ^**^p < 0.01, *ns*, not significant.

To supplement these unexpected results, we next performed unplanned exploratory tests directly comparing high frequency spectral power in participants who were asymptomatic-to-low levels of symptomatology (defined as a score of 10 or less on the CAPS, *n* = 28) with those exhibiting moderate-to-severe symptoms (defined as a score of >22 on the CAPS, *n* = 44). NREM high frequency power was defined as PSD averaged between 20.31 and 29.88 Hz. REM high frequency power was defined as PSD averaged between 23.24 and 29.88 Hz (i.e., we averaged across frequencies in the significant clusters that emerged in our primary analyses; Figures 1, 2). In this analysis, high frequency spectral power was significantly higher in asymptomatic-to-mild symptoms participants compared to moderate-to-severe participants for both NREM [*t*_(39.3)_ = 2.74, *p* = 0.01, *d* = 0.70] and REM [*t*_(38.2)_ = 2.79, *p* = 0.009, *d* = 0.72] sleep (Figure 3A). Within-groups, there were no significant correlations with either PCL or CAPS hyperarousal scores (all *p'*s > 0.063) or PCL or CAPS total score (all *p'*s > 0.16).

Heightened spectral power in the 20–30 Hz range has previously been linked to hyperarousal in insomnia patients. Although the current dataset lacked a validated measure of insomnia symptoms, we were able to examine how spectral power differed between generally good and poor sleepers, as defined using the Pittsburgh Sleep Quality Index (PSQI). As an unplanned exploratory analysis, we divided the sample into participants with either good or poor sleep quality. Those with good sleep quality were defined as participants with a PSQI score in the bottom two quintiles of the distribution of PSQI scores (*M* = 3.96, *SD* = 1.36, *n* = 27). Those with poor sleep were defined as participants with a PSQI score in the top two quintiles (*M* = 10.6, *SD* = 1.78, *n* = 28). We again defined high frequency power by averaging across frequencies in the significant clusters that emerged in our primary analyses (Figures 1, 2). We did not see any differences between good and poor sleepers with regards to either NREM [*t*_(52)_ = 0.93, *p* = 0.35, *d* = 0.25] or REM [*t*_(48.2)_ = 0.83, *p* = 0.41, *d* = 0.23] spectral power (Figure 3B).

**Figure 4:**
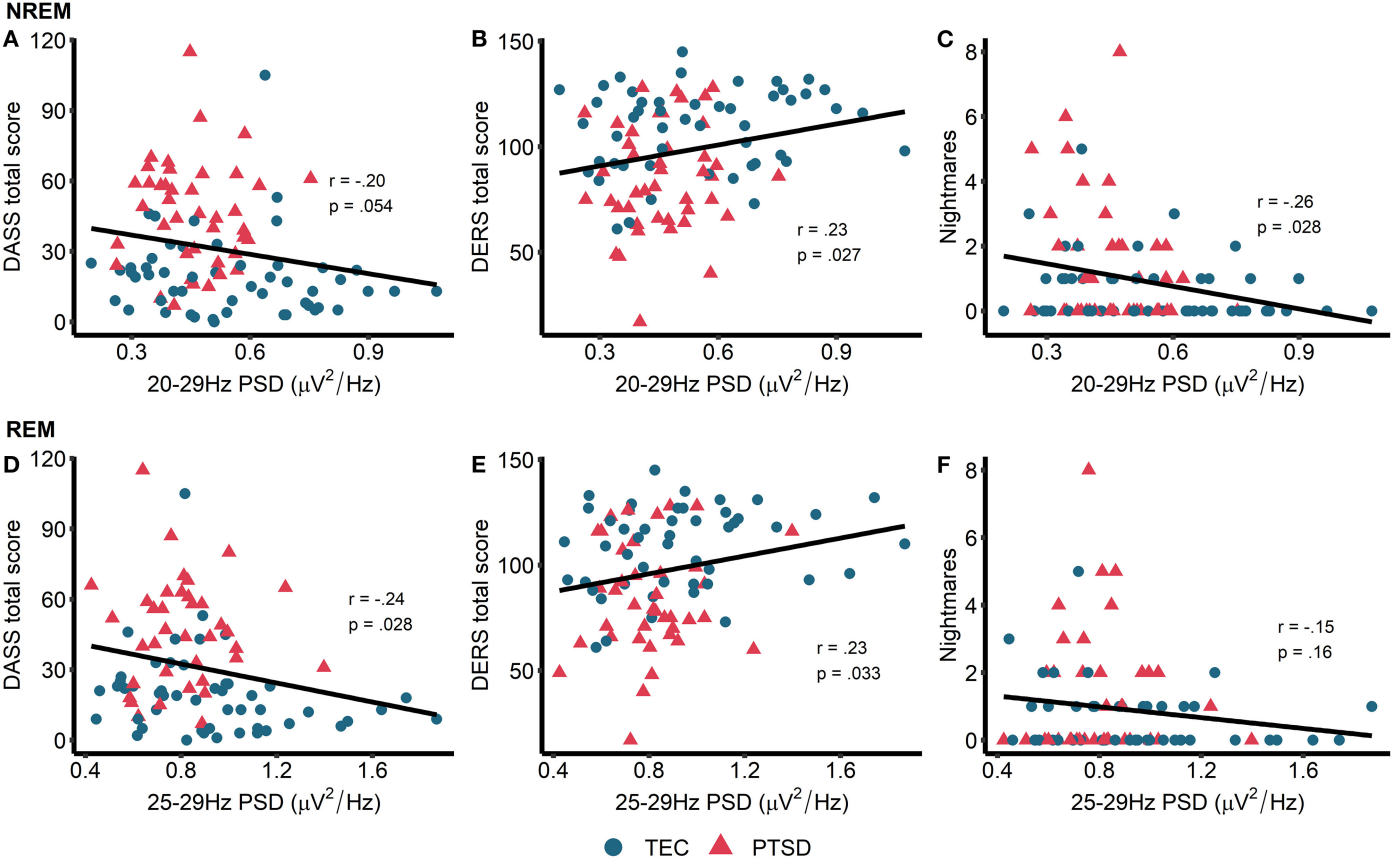
Exploratory correlations. Top row: correlations between NREM high frequency power and general psychopathology (higher score = more symptoms of depression, anxiety, and stress) **(A)**, emotion regulation (higher score = better able to regulate emotions) **(B)**, and nightmare frequency **(C)**. Bottom row: correlations between REM high frequency power and general psychopathology **(D)**, emotion regulation **(E)**, and nightmare frequency **(F)**. Note that PSD at each frequency is relative to the average PSD in the 0–30 Hz range.

This set of results, that high frequency spectral power is *inversely* associated with PTSD symptomatology, directly contradicting our original hypotheses. To better understand these findings, we next performed unplanned exploratory correlations between high frequency PSD and other measures of psychopathology and well-being. In particular, we focused on symptoms of depression, anxiety, and stress (as measured by the DASS), ability to regulate emotions [as measured by the DERS, on the basis of other research linking oscillatory EEG activity during wake is related to emotion regulation PTSD; (51)] and nightmare frequency [on the basis that nightmares in PTSD have been theorized to reflect hyperarousal; (52, 53)]. High frequency power during NREM sleep showed a trend toward being associated with lower overall DASS-measured psychopathology (*r* = −0.20, *p* = 0.054) along with significantly better emotion regulation (*r* = 0.23, *p* = 0.027) and significantly fewer nightmares (*r* = −0.26, *p* = 0.011; Figure 4). For REM sleep, high frequency PSD was associated with lower overall psychopathology (*r* = −0.24, *p* = 0.028) and better emotion regulation (*r* = 0.23, *p* = 0.033; Figure 4). No other correlations were significant.

### Sleep Spindles

**Figure 5:**
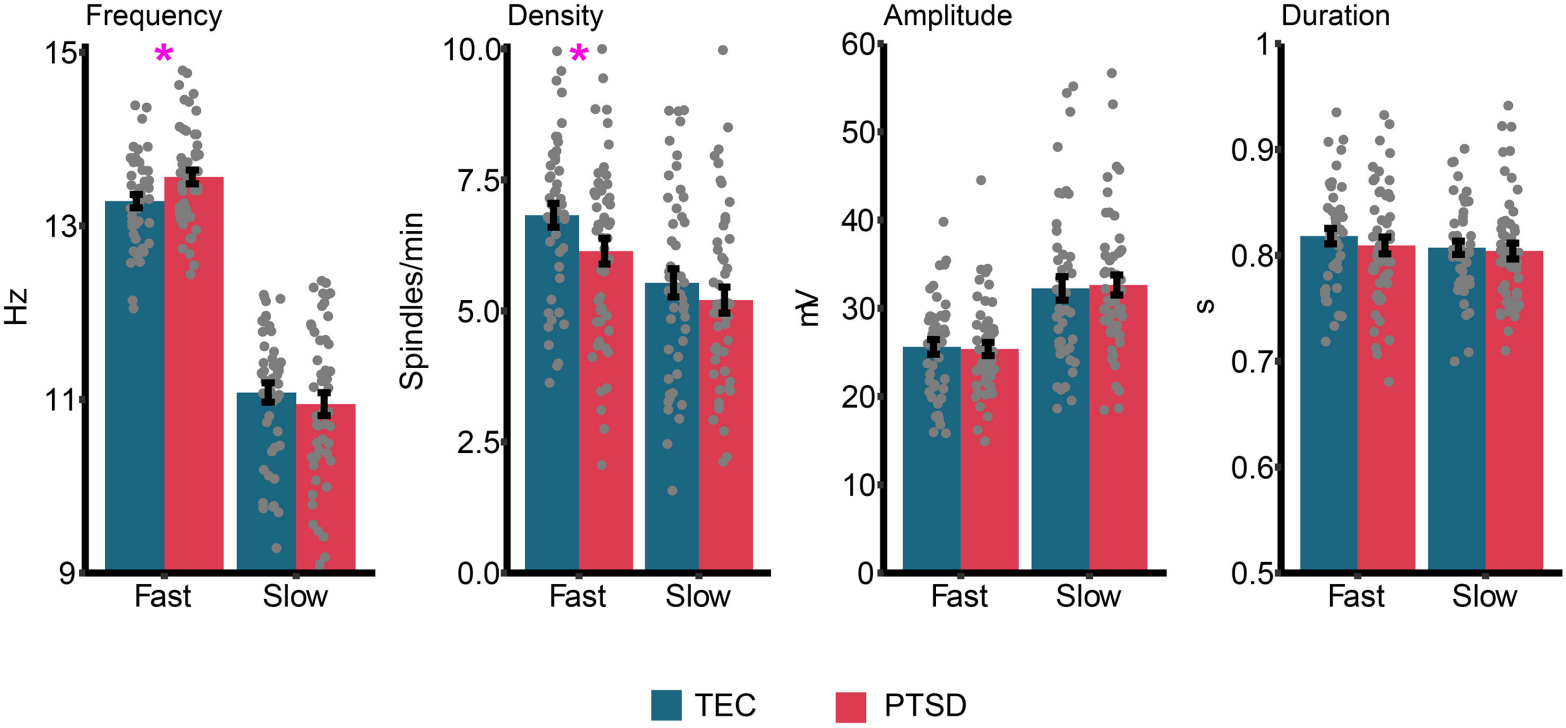
Group differences in sleep spindle properties. Gray dots represent individual data points, and error bars indicate the standard error. ^*^p < 0.05.

Group differences in sleep spindle properties are displayed in Figure 5. Our pre-registered *a priori* hypothesis was that sleep spindle peak frequency would be faster in PTSD patients compared to TEC. For fast spindles, a small but significant difference was observed [*t*_(94.94)_ = 2.51, *p* = 0.014, *d* = 0.51] with spindle peak frequency being slightly faster in PTSD (*M* = 13.56 Hz, *SD* = 0.57 Hz) compared to TEC (*M* = 13.28 Hz, *SD* = 0.52 Hz). No difference in peak frequency was found for slow spindles [*t*_(94.53)_ = 0.77, *p* = 0.44, *d* = 0.15]. The same pattern of results were observed when fast spindle frequency was examined during N2 [*t*_(94.87)_ = 2.52, *p* = 0.013, *d* = 0.51], N3 [*t*_(94)_ = 2.31, *p* = 0.023, *d* = 0.47], early NREM [*t*_(95)_ = 2.53, *p* = 0.013, *d* = 0.51], or late NREM [*t*_(94.84)_ = 2.52, *p* = 0.016, *d* = 0.50] sleep. One possibility is that a higher spindle frequency is a by-product of faster EEG activity. To test this hypothesis, we correlated fast spindle frequency with high frequency power (20.31–29.88 Hz, frequencies derived from our primary analysis; see Power spectral density results). We did not find a significant correlation either across all participants (*r* = −0.16, *p* = 0.12), or when TEC (*r* = −0.09, *p* = 0.51) and PTSD (*r* = −0.21, *p* = 0.17) were assessed separately.

We next ran planned exploratory tests on other spindle properties. The only significant difference (uncorrected) was reduced fast spindle density in PTSD (*M* = 6.14, *SD* = 1.75) relative to TEC [*M* = 6.82, *SD* = 1.54; *t*_(94.94)_ = 2.05, *p* = 0.043, *d* = 0.42]. We note that the difference in fast spindle density was no longer significant after controlling for multiple comparisons. All other comparisons were non-significant (all *p*'s > 0.36, all *d*'s <0.19). Correlations between spindle properties and PTSD symptomatology are shown in Table 2. The only (uncorrected) significant results were a positive correlation between slow spindle amplitude and symptomatology.

**Table 2:** Correlations between sleep spindle properties and symptomatology.

	**PCL hyperarousal**	**PCL total**
Fast spindle frequency	*r* = 0.09 *p* = 0.38	*r* = 0.05 *p* = 0.66
Slow spindle frequency	*r* = −0.08 *p* = 0.44	*r* = −0.11 *p* = 0.27
Fast spindle density	*r* = −0.10 *p* = 0.32	*r* = −0.14 *p* = 0.16
Slow spindle density	*r* = 0.06 *p* = 0.53	*r* = 0.03 *p* = 0.80
Fast spindle amplitude	*r* = 0.06 *p* = 0.53	*r* = 0.13 *p* = 0.19
Slow spindle amplitude	***r*** **=** **0.24** ***p*** **=** **0.02**	***r*** **=** **0.25** ***p*** **=** **0.01**
Fast spindle duration	*r* = −0.09 *p* = 0.38	*r* = −0.11 *p* = 0.29
Slow spindle duration	*r* = 0.04 *p* = 0.70	*r* = 0.12 *p* = 0.23

*Bold values indicate cases where p < 0.05 (uncorrected)*.

## Discussion

In this report, we investigated the relationship between sleep microarchitecture (power spectral density and spindles) and psychopathology symptom severity in trauma-exposed participants with and without diagnosed PTSD. Contrary to our hypothesis, we observed elevated beta spectral power activity for TEC compared to PTSD participants during both NREM and REM sleep. We did not replicate prior reports implicating REM theta power for PTSD resilience. Beta power was further associated with reduced hyperarousal, decreased nightmare frequency, decreased depression, anxiety and stress, and better ability to regulate emotions. Interestingly, we also observed fewer, but higher frequency fast sleep spindles in PTSD compared to TEC participants while slow spindle amplitude was positively associated with hyperarousal. The following sections aim to interpret these findings.

### Beta Spectral Power Decreased in PTSD

High frequency EEG rhythms (e.g., beta rhythms) during sleep are theorized to be a component of a hyperarousal syndrome present in Insomnia Disorder [(54–60); though see (61) who failed to detect this in a large meta-analytic study], a condition commonly comorbid with anxiety and traumatic-stress disorders, such as PTSD. However, associations between elevated beta power and PTSD symptomatology remains mixed. For example, in PTSD compared to non-PTSD controls, some studies have observed higher waking beta EEG power (62), higher beta power across a whole night of sleep (8, 9), higher REM-NREM sleep beta power ratios (12), and greater high frequency activity (including beta power) during NREM sleep (63). Nonetheless, others have failed to observe such group differences (13, 14).

Here, we surprisingly observed that beta power was increased in trauma-exposed non-PTSD participants compared to participants that met criteria for PTSD. Inconsistencies among studies of sleep beta activity in PTSD may be explained by a number of factors. First, it is unclear whether beta power represents a biomarker of trauma exposure as opposed to a marker of hyperarousal or PTSD severity. In support of the former interpretation, NREM beta power has been shown to be positively associated with prior combat exposure in those with PTSD (13). Second, beta power may be an indicator of adaptive emotional processing (64). For example, a prospective study linked increased REM beta power soon after a trauma to decreased PTSD and nightmare severity at a follow-up 2 months later (14). We similarly observed a negative association between beta power and PTSD symptomatology, including self-reported hyperarousal and nightmare frequency, albeit during NREM sleep. These associations gain convergent validity by the fact that the DASS, DERS, and nightmare frequency varied with PTSD symptomatology in the same manner. To our knowledge, no prospective studies have assessed whether beta power is a trait vulnerability factor or a marker of PTSD pathogenesis. While we recognize such a study might be difficult to perform, others have studied baseline sleep and subsequent PTSD development in groups with a high likelihood of experiencing subsequent trauma [e.g., military personnel; (65)]. Third, studies to date differ as to when sleep was measured relative to the index trauma, with some measuring sleep in close proximity to the traumatic event [e.g., (14)] and others measuring sleep decades after trauma exposure [e.g., (12)]. We report here associations between current sleeping patterns and psychopathology in individuals with a relatively recent (1 month to 2 years) index trauma. Future work might focus on prospectively mapping trajectories of sleep microarchitecture over the course of PTSD development and maintenance.

Another potential reason for heterogeneity among prior reports may be that whereas beta power may be a marker of insomnia severity, a sleep disorder commonly comorbid to PTSD and associated with a daytime hyperarousal syndrome (66), it may not reflect PTSD-specific hyperarousal over and above trauma exposure itself. While we did not directly measure insomnia severity in the present study, we did not find that poorer sleep quality (measured by comparing widely separated PSQI scores) were associated with greater beta power. Interestingly, a prospective treatment study for patients experiencing insomnia found that improvements in psychopathology and insomnia severity were associated with increased beta power during NREM sleep (64). Contrary to earlier theories (58), beta power appears to be adaptive to regulating emotions or learning emotion control techniques. Further, other studies have failed to find an association between beta power during sleep and insomnia symptomatology (63) or subjective hyperarousal symptoms (12). Nonetheless, current evidence is far from definitive and requires further exploration.

Lastly, several studies to date likely lack the power to detect consistent sleep microarchitectural features related to PTSD symptomatology owing to small sample sizes, and underpowered studies are highly susceptible to Type 2 error (67). To our knowledge, our study constitutes the largest sample size to date that spans the full spectrum of post-traumatic stress symptomatology. Increasing power through meta-analytic techniques is a practical and important next step to update current trends in the literature related to sleep spectral power and PTSD symptomatology.

### Sleep Spindle Frequency Differences in PTSD

Here, we report increased fast spindle frequency during NREM sleep in PTSD compared to TEC. This aligns with recent reports of sleep spindle morphology differences in PTSD (6, 21, 63). We found that spindle peak frequency was not correlated with activity at faster frequencies (>20 Hz), suggesting that the group difference in spindle frequency was independent to EEG activity at faster frequencies.

While we did not find that these group differences predicted symptom severity, other reports have found that spindle activity predicted daytime intrusive symptoms (63) and fragmented sleep (21). Interestingly, several other reports have linked NREM sigma power (the frequency band encompassing sleep spindles) with increased susceptibility to post-traumatic symptomatology in rodent models (68) and humans (12, 69). Specifically, human studies have shown a positive association between NREM sigma activity and subjective hyperarousal (12) as well as intrusive symptoms (69) in those with PTSD.

While speculative, fast spindle activity, when coupled with cortical slow oscillations and hippocampal sharp-wave ripples, has been linked to enhanced consolidation of fear memories in rodent models of PTSD (70). This aligns with targeted memory reactivation studies in humans showing that fear memory cueing during post-learning NREM sleep alters subsequent conditioned-fear responses (71, 72). Moreover, consolidation of extinction memories may be impaired when fear reminders are presented during slow wave sleep following extinction learning (73). However, like beta power, it remains unknown whether changes in spindle morphology in PTSD is an indicator of disorder pathogenesis or a trait vulnerability factor. While spindle activity has been shown to be stable across multiple nights of sleep (37), associative learning (including fear conditioning) has been shown to increase NREM sleep post-learning in rodents (74) and sleep spindle density in humans (32). An important next step will be to determine whether spindle activity alone, or spindles specifically coupled with slow oscillations [which have been predictive of emotional memory consolidation; e.g., (31, 70, 75, 76)], alters the processing of conditioned fear and extinction memories both in healthy participants as well as those with anxiety and traumatic stress disorders.

### Limitations

The current study was limited in a number of ways. First, the study is cross-sectional, including analysis of only a single night of recorded sleep. We therefore could not investigate some key questions addressed above including whether specific sleep oscillatory rhythms are trait vulnerability factors, diagnostic of PTSD disorder sequelae or a combination of the two. We limited our analysis to a single night in order to reduce potential confounds from first night effects (i.e., not analyzing the acclimation/diagnostic recording) and learning (i.e., not analyzing the post-fear conditioning night). Second, our sleep recordings took place in participants' own homes, reducing experimental control. However, we believe this approach was a strength as such recordings may be more indicative of the typical sleep our participants obtain on a nightly basis. Thirdly, the stored PSG files limited our ability to look beyond 30 Hz activity, into the gamma frequency range. Gamma activity during sleep has been associated with reduced overnight emotional processing [as indexed by behavioral responses and functional brain activity in emotional brain regions; (77)] and has been shown to be increased in PTSD (6), and could be an interesting range to investigate in future research. Fourthly, because antidepressant treatment is extremely common among those with moderate to severe PTSD, individuals receiving a stable dose of an antidepressant were accepted into the study on a case-by-case basis and these drugs may have influenced EEG spectral power in certain participants.

### Conclusions

In a large sample of trauma-exposed participants expressing a wide range of post-traumatic symptomatology, we found NREM beta power to be decreased and fast spindle peak frequency increased in participants meeting criteria for PTSD compared to trauma-exposed participants without PTSD. Contrary to several prior reports, beta power was associated with better, rather than poorer mental health on a variety of measures. Whether these sleep rhythms may be protective from PTSD pathogenesis remains to be determined.

## Data Availability Statement

The data analyzed in this study is subject to the following licenses/restrictions: the data underlying this article will become available in the future in the NIMH Data Archive (NDA) at https://nda.nih.gov, and can be accessed following instructions at https://nda.nih.gov/get/access-data.html. Requests to access these datasets should be directed to https://nda.nih.gov/get/access-data.html.

## Ethics Statement

The studies involving human participants were reviewed and approved by Massachusetts General Hospital Internal Review Board. The patients/participants provided their written informed consent to participate in this study.

## Author Contributions

DD, RB, TC, SZ, and EP-S conceived the hypotheses and analysis plan. DD analyzed the data. RB and DD wrote the paper. TC, SZ, and EP-S read and edited drafts of the paper. CD, KO, KM, and SG collected the data. CD, KO, KM, SG, AK, and UM carried out data reduction, management, and initial processing. KG performed the sleep scoring. NL performed the diagnostic clinical interviews. All authors contributed to the article and approved the submitted version.

## Funding

This project was supported by NIMH grants R01MH109638 and R21MH115279 to EP-S. RB and TC were funded by the Research Training Program in Sleep, Circadian and Respiratory Neurobiology (NIH T32 HL007901) through the Division of Sleep Medicine at Harvard Medical School and Brigham & Women's Hospital. Research was carried out at the Athinoula A. Martinos Center for Biomedical Imaging, Charlestown MA, and the Massachusetts General Hospital, Department of Psychiatry, Psychiatric Neuroimaging Division.

## Conflict of Interest

Praxis Precision Medicines, Inc. provides partial salary support to EP-S. The remaining authors declare that the research was conducted in the absence of any commercial or financial relationships that could be construed as a potential conflict of interest.

## Publisher's Note

All claims expressed in this article are solely those of the authors and do not necessarily represent those of their affiliated organizations, or those of the publisher, the editors and the reviewers. Any product that may be evaluated in this article, or claim that may be made by its manufacturer, is not guaranteed or endorsed by the publisher.
